# Cockayne Syndrome B protein antagonizes OGG1 in modulating CAG repeat length *in vivo*

**DOI:** 10.18632/aging.100324

**Published:** 2011-05-03

**Authors:** Irina V. Kovtun, Kurt O. Johnson, Cynthia T. McMurray

**Affiliations:** ^1^ Department of Pharmacology and Experimental Therapeutics, Mayo Clinic and Foundation, Rochester, MN 55905, USA; ^2^ Life Sciences Division, Lawrence Berkeley National Laboratory, Berkeley, CA 94720, USA

**Keywords:** CSB, transcription coupled repair, CAG expansion, base excision DNA repair, Huntington's Disease

## Abstract

OGG1 and MSH2/MSH3 promote CAG repeat expansion at Huntington's disease (HD) locus *in vivo* during removal of oxidized bases from DNA. CSB, a transcription-coupled repair (TCR) protein, facilitates repair of some of the same oxidative lesions. *In vitro*, a knock down CSB results in a reduction of transcription-induced deletions at CAG repeat tract. To test the role of CSB *in vivo*, we measured intergenerational and somatic expansion of CAG tracts in *HD* mice lacking CSB, OGG1, or both. We provide evidence that CSB protects CAG repeats from expansion by either active reduction of the tract length during parent-child transmission, or by antagonizing the action of OGG1, which tends to promote expansion in somatic cells. These results raise a possibility that actions of transcription-coupled and base excision repair pathways lead to different outcomes at CAG tracts *in vivo*.

## INTRODUCTION

The progression and severity of triplet repeat disorders are exquisitely sensitive to the size of the triplet tract length [1-3]. Thus, there has been intense interest in determining whether attenuation of tract expansion or promotion of tract deletion has therapeutic potential. In this regard, DNA repair proteins have been the focus of much attention [1-3]. In mice, loss of Ku, Rad 52, Rad 54 [4], Xeroderma pigmentosum Type C (XPC) [5], MSH6 [6, 7], alkyladenine DNA glycosylase [AAG] [8], endonuclease III (NTH) [8] have little effect on changing CAG or CTG tract length, indicating that double strand break repair, global genomic nucleotide excision repair (GG-NER), and repair of single base mismatches have little influence on the change in tract size. On the other hand, the mismatch repair (MMR) pathway for small loops appears to promote expansion. Loss of MMR proteins MSH2 [4-6, 9-13] and MSH3 [6, 7], specifically attenuates CAG/CTG expansion in somatic and germ cells. In the absence of MSH2, the length of the CAG/CTG tracts become increasingly smaller as the genes are passed down from generation to generation [11, 12]. Loss of PMS2 [14], the downstream binding partner of MMR recognition complexes, has significantly smaller effects on CAG/CTG instability relative to loss of MSH2/MSH3.

Recently, we have reported that loss of a base excision repair (BER) protein 7,8-dihydro-8-oxoguanine glycosylase (OGG1) significantly diminishes CAG expansion in HD mice [8]. We have speculated that the BER and MMR pathways cooperate to cause expansion during an attempt to repair 7,8-dihydro-8-oxoguanine (8-oxoG) lesion [8]. OGG1 is a main glycosylase responsible for recognition and removal of 8-oxoG. Mice lacking OGG1 show accumulation of 8-oxoG lesions and increase in the level of spontaneous mutation frequency [15-17]. However, detailed analyses in these studies also indicated that repair of 8-oxoG in *OGG1*(−/−) cells was not completely abolished suggesting a back up for the repair of this lesion in the absence of OGG1. A number of studies have examined the role of key transcription-coupled repair (TCR) protein, Cockayne's Syndrome B (CSB), in removal of oxidative damage. Embryonic fibroblasts from *CSB*(−/−) mice were reported to be more sensitive to compounds which induce oxidative stress [18]. Both nuclear and mitochondrial DNA have been tested for accumulation of various oxidative lesions in the absence of functional CSB protein. The rates of accumulation of 8-oxoG and 8-oxoA in nuclear DNA were higher in CSB-deficient cells [19, 20] than in wild type cells. Similarly, both CSB-null and helicase motif V/VI mutant cells showed a lower activity for 8-oxoG incision in nuclear DNA than cells carrying a functional CSB [21, 22]. Lower incision level for 8-oxoG in CS cells correlated with reduced expression level of OGG1 [22]. A more recent reports demonstrated that CSB protein localizes to mitochondria, and the level of CSB in this cellular compartment is elevated upon oxidative stress [23, 24]. Following treatment with hydrogen peroxide CSB was detected inside of mitochondria in the complex with mtDNA, OGG1 and single-stranded DNA binding protein [24]. Based on these data CSB has been suggested to play a direct role in mitochondrial BER by facilitating recruitment and stability of DNA repair complexes inside of mitochondria [23]. Likewise, in tissues of CSB knockout mice such as kidney, brain and liver elevated levels of FapyA and FapyG lesions, substrates for NEIL1 DNA glycosylase, were detected (25). *In vitro*, CSB stimulated enzymatic activity of NEIL1 (26) and has been shown to interact with other BER enzymes, including AP endonuclease 1 [26] and poly(ADP-ribose)polymerase (PARP1) [27]. A role of CSB in global repair of oxidative damage has been demonstrated *in vivo* using a mouse that carried non-transcribed bacterial gene *lacI* [28]. Comparison of mutation frequencies at *lacI* between *OGG1*(−/−), *CSB^m/m^*, (mice which contain severely truncated version of CSB gene) and *CSB^m/m^OGG1(−/−)* animals has revealed an elevated level in the latter suggesting that CSB is involved in the inhibition of mutations caused by oxidative DNA damage in a non-transcribed gene [28].

A role of TCR-NER proteins and transcription in stability of CAG/CTG repeats has been examined in several model systems. The destabilizing effect of transcription alone on repeat tract was reported in bacteria, yeast and flies [29]. CAG/CTG tracts were shown to delete upon active transcription in human cell lines carrying long CAG repeats within intronic sequence of HPRT minigene [29]. siRNA knockdown of CSB in these cell lines reduces the frequency of the deletions [30]. Similarly, the transcription-induced germline instability of expanded CAG repeat locus within SCA3 transgene was significantly diminished in germ cells of *Drosophila* lacking a homolog of XPG, another key enzyme of TC-NER pathway [31]. The role of TC-NER in trinucleotide stability in mice *in vivo* has not been examined. Whether CSB contributes to repeat expansion by facilitating repair of oxidized bases within CAG tract has not been tested in mice. In order to address this we generated a cross between *HD* transgenic mouse R6/1 and a mouse knock out for CSB protein. In these crosses we have analyzed changes in CAG tract i) upon parent to progeny transmission and ii) in somatic tissues upon aging. Our results here indicate that the deletion of CSB leads to expansion exacerbation in the germ cells. The level of expansion is largely unchanged in somatic tissues of *CSB*(−/−) mice as compared to R6/1 counterparts, while *CSB/OGG1* double knock out mice show further progression of somatic expansion with age, effect opposite to that caused by the deletion of OGG1.

## RESULTS AND DISCUSSION

**Figure 1.**
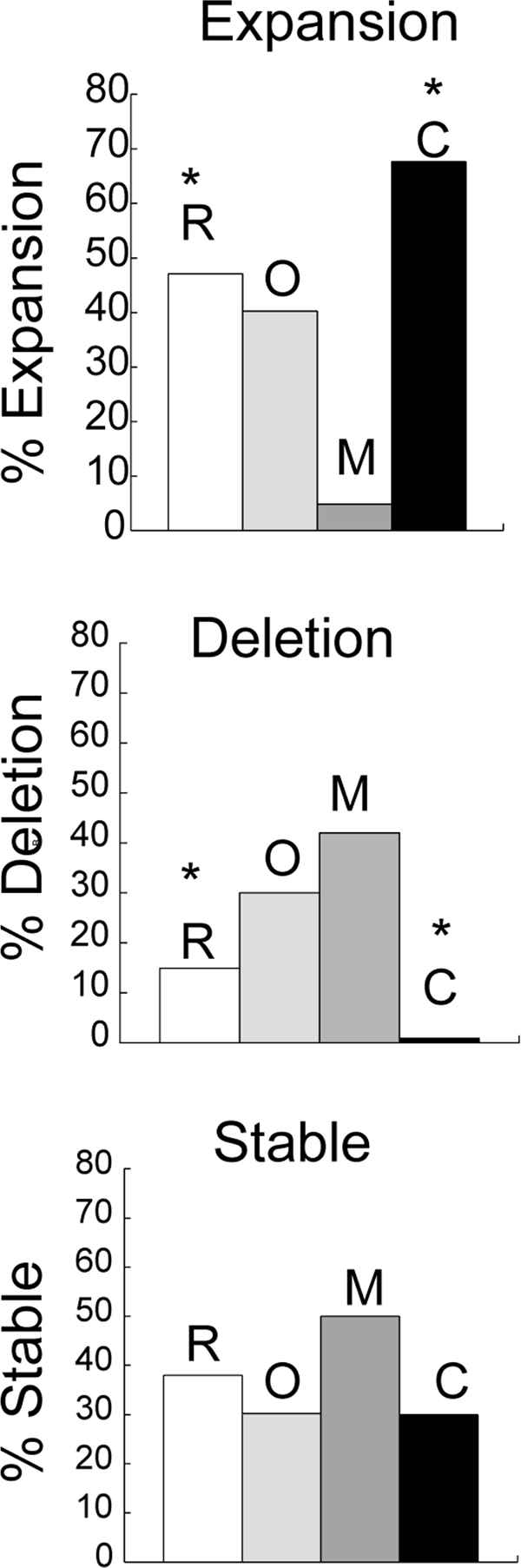
Intergenerational instability in HD mice deficient in different DNA repair enzymes. Bars represent the percentage of progeny that display no change (stable), expansion or deletion of the CAG repeats in mice that harbor the human *HD* transgene in R6/1 **(R)**, n=8 (white); *R6/1*(−/+)/*Ogg1(−/−)* **(O)**, n=8 (light grey), *R6/1D*(−/+)/*Msh2(−/−)* (**M**), n=12 (dark grey) and *R6/1*(−/+)/*CSB(−/−)* (**C**), n=10 (black). Genescan analysis was performed on tail DNA at 3 weeks of age. Repeat length changes ranged from −4 to +4. The asterisk indicates statistically significant differences in expansions and deletions as judged by Student's *t*-test (*P*<0.05).

To test the role of CSB on expansion *in vivo*, we crossed *R6/1* mice [32]harboring an expanded number (130-140 units) of CAG repeats in exon 1 of human *HD* gene (130-140 CAGs) with mouse lacking CSB protein [33]. The *HD* transgene is ubiquitously expressed in tissues of R6/1 mice [32], and CAG repeats at the *HD* locus expand upon repair of oxidative damage [8]. We analyzed both somatic and intergenerational changes in CAG tract length in the *R6/1/CSB*(−/−) crosses. In the germ cells, when compared to their *R6/1* counterparts (Figure 1, deletion, white bar) the per cent of deletions in *CSB*(−/−) animals was low (Figure 1, deletion, black bar) and the per cent of stable transmissions remained the same (Figure 1, stable, compare white and black bars). Remarkably, however, *CSB*(−/−) animals showed a 5-fold increase in expansion (Figure 1, expansion, black bar). In fact, CSB promoted reduction of the CAG tract size during transmission *in vivo* as effectively as MSH2 promoted expansion (Figure 1, compare dark grey and black). The absence of OGG1 did not significantly affect the level of intergenerational instability (Figure 1, light grey), suggesting that germ cells did not accumulate a sufficiently high levels of oxidative lesions as did the aging somatic cells, where OGG1 was shown to play a more substantial role [8]. Thus, CSB plays a protective role by reducing the CAG tract size during parent-child transmission through the germline.

**Figure 2.**
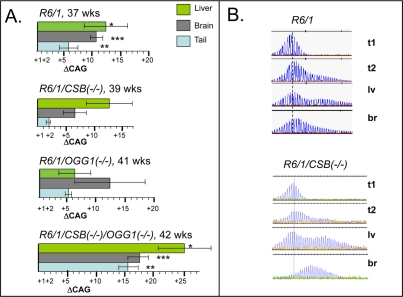
Somatic instability in HD mice deficient in different DNA repair enzymes. (**A**) Age-dependent somatic expansion in selected tissues of *R6/1, R6/1(−/+)/CSB(−/−), R6/1(−/+)/OGG1(−/−), R6/1(−/+)/CSB(−/−)/OGG1*(−/−) presented as the mean length change of CAG repeats and SD. Ages and tissues are indicated. The asterisks indicate statistically significant differences as judged by Student's *t*-test; P<0.01 for (*), (**) and (***). (**B**) CAG repeat distributions in somatic tissues of control *R6/1* and CSB knock out mice. t1 is repeat distribution in tail at 3 weeks; while expansion at 39-42 weeks in shown in tail (t2), brain (br) and liver (lv). Vertical dashed line designates the major CAG length in the distributions measured in tail DNA at 3 weeks of age.

We then examined changes in CAG tract lengths in somatic tissues of aging *CSB(−/−)* mice and compared them to *R6/1* and *OGG1*(−/−) mice. Expansion was previously shown to be attenuated in *R6/1/MSH2(−/−),R6/1/MSH3(−/−)*, and suppressed in 70% of *R6/1/OGG1*(−/−) animals. In contrast, at 39 weeks of age, expansion in liver and brain at the *HD* locus in *R6/1 /CSB*(−/−) mice did not diminish and was similar to that of *R6/1* animals (Figure 2). Since both OGG1 and CSB are involved in repair of oxidative lesions, and OGG1 tended to promote CAG expansion in somatic cells [8], we tested whether CSB and OGG1 had genetic interactions that affected the CAG tract length (Figure 2). We created *R6/1/CSB(−/−)/OGG1*(−/−) double knockout mice, and compared the level of instability in somatic tissues relative to *R6/1* and each knockout alone. Opposite to expected, we found that expansion in liver, brain and even tail in *R6/1/CSB(−/−)/OGG1(−/−)* mice has progressed with age more than in corresponding tissues of *R6/1* or each single knock out animals.

Thus, CSB appeared to antagonize and override the effects of OGG1 *in vivo*, and exert a stabilizing effect on the CAG tract in aging somatic cells. The CSB-OGG1 antagonism at the CAG tract is surprising as both proteins are involved in repair of oxidative damage and appear to act in the same pathway. On the other hand, CSB has been described to have additional functions. It is known to have chromatin remodeling activity [34] and to affect transcription [35]. Role of CSB in transcription regulation is not limited to damage-containing loci. The protein is reported to localize to active transcription sites in the absence of DNA damage [36], to associate with complexes containing RNA polymerase I, II and III and to stimulate polymerase elongation activity [37-39]. *In vitro*, CSB has been shown to interact with both double stranded DNA and core histones to modulate nucleosome structure [34].

It was also reported to directly regulate transcription of the genes involved in chromatin remodeling in the absence of external stress [40]. Although recent studies have identified the exact domains within CSB that are responsible for its repair [41] and chromatin remodeling activities [42], the details of how different functions of CSB are integrated are not well understood. In relation to the role of CSB in controlling CAG repeat stability, CSB may facilitate chromatin re-arrangements and coordinate the action of transcription and/or repair machinery at the transcription sites or repair sites, thereby keeping repeat length in check. It is possible that in the absence of CSB, the stalled at the CAG repeats polymerase transcription complex is processed in a chromatin context in a way that promotes the formation of hairpins [3], resulting ultimately in bigger expansions. Future studies are needed to elucidate the mechanism by which CSB regulates stability of triplet repeats.

In conclusion, our results here suggest that the mechanism by which CSB is influencing CAG repeat stability is different from that of OGG1 and may involve its chromatin maintenance/remodeling activity.

## MATERIALS AND METHODS

### Mouse lines and breeding

Transgenic male mice B6CBA-TgN R6/1 [32] were crossed with C57BL/6J female partners that lacked one of the glycosylases OGG1 [8], or TCR-NER protein CSB and bred until they were homozygous knockout. Litters were screened for the presence of human *HD* transgene [ref. 10 and below] and the absence of each glycosylase by PCR using following primers: 1) for OGG1- ATGAGGACC AAGCTAGGTGAC (common), GCCTCACAATCAA CTTATCCC (wild type) and ATCTGCGTGTTCGAA TTCGCCAAT (knock out); 2) for CSB-GCTG CTTA TAATAATCCTCATCTCC (common), ATCTGCGT GTTCGAATTCGCCAATG (knock out) and GTCTT CTGATGACGTTAGCTATGAG (wild type), 3) for NTH1 TCCCACTTTGTGTTCTAAGTACTGGGT and GTCACAGACGCGACCATACTC ATTAG-knock out; and CCTTGGCTCCTGCATGTGCCCAAT and GCCAGAAGGGCTTCCTATGCATTTGTGGT-wild type.

### GeneScan analysis of CAG repeats

DNA was prepared from mouse tissues at the indicated age 70 ng of DNA was amplified with specific primers 1, 5'–AAAAGCT GATGAAGGCCTTCGAG–3',-labeled with a fluorescence tag, propyl-phos phoramidite (6-FAM) (Glen Research) and 2, 5'–CGGCGGTGGCGGCTGT TG–3') as described [10]. An internal size standard (2500-TAMPA (Perkin Elmer)) was added to a 1.0 μl aliquot of amplified fluorescent product the products were analyzed using ABI prism 3700 DNA analyzer instrument and the GeneMapper software v3.

### Statistical analysis

The repeat size of the offspring (selected as the size of the major CAG length in the distributions) in each knock out group of mice was compared to those in wild type group using a *t* test. P value for a given sample number is presented (Figure 1).

## References

[R1] Mirkin SM (2007). Expandable DNA repeats and human disease. Nature.

[R2] Pearson CE, Edamura NK, Cleary JD (2005). Repeat instability: mechanisms of dynamic mutations. Nat Rev Genet.

[R3] Kovtun IV, McMurray CT (2008). Features of trinucleotide repeat instability in vivo. Cell Res.

[R4] Savouret C, Garcia-Cordier C, Megret J, te Riele H, Junien C, Gourdon G (2005). MSH2-dependent germinal CTG repeat expansions are produced continuously in spermatogonia from DM1 transgenic mice. Mol Cell Biol.

[R5] Dragileva E, Hendricks A, Teed A, Gillis T, Lopez ET, Friedberg EC, Kucherlapati R, Edelmann W, Lunetta KL, MacDonald ME, Wheeler VC (2009). Intergenerational and striatal CAG repeat instability in Huntington's disease knock-in mice involve different DNA repair genes. Neurobiol Dis.

[R6] van den Broek WJ, Nelen MR, Wansink DG (2002). Somatic expansion behaviour of the (CTG)n repeat in myotonic dystrophy knock-in mice is differentially affected by Msh3 and Msh6 mismatch-repair proteins. Hum Mol Genet.

[R7] Owen B.A., Badger J.D., Yang Z., Lai M., Hayes J.J., Wilson T., Edelman W., Kucherlapati R, McMurray CT (2005). (CAG)(n)-hairpin DNA binds to Msh2-Msh3 and changes properties of mismatch recognition. Nat Struct Mol Biol.

[R8] Kovtun IV, Liu Y, Bjoras M, Klungland A, Wilson SH, McMurray CT (2007). OGG1 initiates age-dependent CAG trinucleotide expansion in somatic cells. Nature.

[R9] Manley K., Shirley TL, Flaherty L, Messer A (1999). Msh2 deficiency prevents in vivo somatic instability of the CAG repeat in Huntington disease transgenic mice. Nat Genet.

[R10] Kovtun IV, McMurray CT (2001). Trinucleotide expansion in haploid germ cells by gap repair. Nat Genet.

[R11] Savouret C, Garcia-Cordier C, Megret J, te Riele H, Junien C, Gourdon G (2004). MSH2-dependent germinal CTG repeat expansions are produced continuously in spermatogonia from DM1 transgenic mice. Mol Cell Biol.

[R12] Kovtun IV, Thornhill AR, McMurray CT (2004). Somatic deletion events occur during early embryonic development and modify the extent of CAG expansion in subsequent generations. Hum Mol Genet.

[R13] Wheeler VC, Lebel L-A, Vrbanac V, Teed A, te Riele H, MacDonald ME (2003). Mismatch repair gene Msh2 modifies the timing of early disease in Hdh(Q111) striatum. Hum Mol Genet.

[R14] Gomes-Pereira M, Fortune MT, Ingram L, McAbney JP, Monckton DG (2004). Pms2 is a genetic enhancer of trinucleotide CAG.CTG repeat somatic mosaicism: implications for the mechanism of triplet repeat expansion. Hum Moll Genet.

[R15] Klungland A, Rosewell I, Hollenbach S, Larsen E, Daly G, Epe B, Seeberg E, Lindahl T, Barnes DE (1999). Accumulation of premutagenic DNA lesions in mice defective in removal of oxidative base damage. Proc Natl Acad Sci USA.

[R16] Minowa O, Arai T, Hirano M, Monden Y, Nakai S, Fukuda M, Itoh M, Takano H, Hippou Y, Aburatani H, Masumura K, Nohmi T, Nishimura S, Noda T (2000). Mmh/Ogg1 gene inactivation results in accumulation of 8-hydroxyguanine in mice. Proc Natl Acad Sci USA.

[R17] Osterod M, Hollenbach S, Hengstler JG, Barnes DE, Lindahl T, Epe B (2001). Age-related and tissue-specific accumulation of oxidative DNA base damage in 7,8-dihydro-8-oxoguanine-DNA glycosylase (Ogg1) deficient mice. Carcinogenesis.

[R18] de Waard H, de Wit J, Gorgels TG, van den Aardweg G, Andressoo JO, Vermeij M, van Steeg H, Hoeijmakers JH, van der Horst GT (2003). Cell type-specific hypersensitivity to oxidative damage in CSB and XPA mice. DNA Repair.

[R19] Balajee AS, Dianova I, Bohr VA (1999). Oxidative damage-induced PCNA complex formation is efficient in xeroderma pigmentosum group A but reduced in Cockayne syndrome group B cells. Nucl Acids Res.

[R20] Tuo J, Jaruga P, Rodriguez H, Bohr VA, Dizdaroglu M (2003). Primary fibroblasts of Cockayne syndrome patients are defective in cellular repair of 8-hydroxyguanine and 8-hydroxyadenine resulting from oxidative stress. FASEB J.

[R21] Tuo J, Müftüoglu M, Chen C, Jaruga P, Selzer RR, Brosh RM, Rodriguez H, Dizdaroglu M, Bohr VA (2001). The Cockayne Syndrome group B gene product is involved in general genome base excision repair of 8-hydroxyguanine in DNA. J Biol Chem.

[R22] Dianov G, Bischoff C, Sunesen M, Bohr VA (1999). Repair of 8-oxoguanine in DNA is deficient in Cockayne syndrome group B cells. Nucl Acids Res.

[R23] Aamann MD, Sorensen M., Hvitby C, Berquist BR, Muftuoglu M, Tian J, de Souza-Pinto NC, Scheibye-Knudsen M, Wilson DM, Stevnsner T, Bohr VA (2010). Cockayne syndrome group B protein promotes mitochondrial DNA stability by supporting the DNA repair association with the mitochondrial membrane. FASEB J.

[R24] Kamenisch Y, Fousteri M, Knoch J, von Thaler AK, Fehrenbacher B, Kato H, Becker T, Dollé ME, Kuiper R, Majora M, Schaller M, van der Horst GT, van Steeg H, Röcken M, Rapaport D, Krutmann J, Mullenders LH, Berneburg M (2010). Proteins of nucleotide and base excision repair pathways interact in mitochondria to protect from loss of subcutaneous fat, a hallmark of aging. J Exp Med.

[R25] Muftuoglu M, de Souza-Pinto NC, Dogan A, Aamann M, Stevnsner T, Rybanska I, Kirkali G, Dizdaroglu M, Bohr VA (2009). Cockayne syndrome group B protein stimulates repair of formamidopyrimidines by NEIL1 DNA glycosylase. J Biol Chem.

[R26] Wong HK, Muftuoglu M, Beck G, Imam SZ, Bohr VA, Wilson DM (2007). Cockayne syndrome B protein stimulates apurinic endonuclease 1 activity and protects against agents that introduce base excision repair intermediates. Nucl Acids Res.

[R27] Thorslund T, von Kobbe C, Harrigan JA, Indig FE, Christiansen M, Stevnsner T, Bohr VA (2009). Cooperation of the Cockayne syndrome group B protein and poly(ADP-ribose) polymerase 1 in the response to oxidative stress. Mol Cell Biol.

[R28] Trapp C, Reite K, Klungland A, Epe B (2007). Deficiency of the Cockayne syndrome B (CSB) gene aggravates the genomic instability caused by endogenous oxidative DNA base damage in mice. Oncogene.

[R29] Lin Y, Hubert L, Wilson JH (2009). Transcription destabilizes triplet repeats. Mol Carcinog.

[R30] Lin Y, Wilson JH (2007). Transcription-induced CAG repeat contraction in human cells is mediated in part by transcription-coupled nucleotide excision repair. Mol Cell Biol.

[R31] Jung J, Bonini N (2007). CREB-binding protein modulates repeat instability in a Drosophila model for polyQ disease. Science.

[R32] Mangiarini L, Sathasivam K, Seller M, Cozens B, Harper A, Hetherington C, Lawton M, Trottier Y, Lehrach H, Davies SW, Bates GP (1996). Exon 1 of the HD gene with an expanded CAG repeat is sufficient to cause a progressive neurological phenotype in transgenic mice. Cell.

[R33] Stevnsner T, Muftuoglu M, Aamann MD, Bohr VA (2008). The role of Cockayne Syndrome group B (CSB) protein in base excision repair and aging. Mech Ageing Dev.

[R34] Citterio E, Van Den Boom V, Schnitzler G, Kanaar R, Bonte E, Kingston RE, Hoeijmakers JHJ, Vermeulen V (2000). ATP-Dependent Chromatin Remodeling by the Cockayne Syndrome B DNA Repair-Transcription-Coupling Factor. Mol Cell Biol.

[R35] Balajee AS, Balajee AM, Dianov GL, Friedberg EC, Bohr VA (1997). Reduced RNA polymerase II transcription in intact and permeabilized Cockayne syndrome group B cells. Proc Natl Acad Sci USA.

[R36] Le May N, Mota-Fernandes D, Vélez-Cruz R, Iltis I, Biard D, Egly JM (2010). NER factors are recruited to active promoters and facilitate chromatin modification for transcription in the absence of exogenous genotoxic attack. Mol Cell.

[R37] Selby Sancar A (2997). Cockayne syndrome group B protein enhances elongation by RNA polymerase II. Proc Natl Acad Sci USA.

[R38] Tantin D, Kansal A, Carey M (1997). Recruitment of the putative transcription-repair coupling factor CSB/ERCC6 to RNA polymerase II elongation complexes. Mol Cell Biol.

[R39] van Gool AJ, Citterio E, Rademakers S, van Os R, Vermeulen W, Constantinou A, Egly JM, Bootsma D, Hoeijmakers JH (1997). The Cockayne syndrome B protein, involved in transcription-coupled DNA repair, resides in an RNA polymerase II-containing complex. EMBO J.

[R40] Newman JC, Bailey AD, Weiner AM (2006). Cockayne syndrome group B protein (CSB) plays a general role in chromatin maintenance and remodeling. Proc Natl Acad Sci USA.

[R41] Anindya R, Mari PO, Kristensen U, Kool H, Giglia-Mari G, Mullenders LH, Fousteri M, Vermeulen W, Egly JM, Svejstrup J.Q (2010). A ubiquitin-binding domain in Cockayne syndrome B required for transcription-coupled nucleotide excision repair. Mol Cell.

[R42] Lake RJ, Geyko A, Hemashettar G, Zhao Y, Fan HY (2010). UV-induced association of the CSB remodeling protein with chromatin requires ATP-dependent relief of N-terminal autorepression. Mol Cell.

